# Larval antlions show a cognitive ability/hunting efficiency trade-off connected with the level of behavioural asymmetry

**DOI:** 10.1007/s10071-018-1190-2

**Published:** 2018-05-14

**Authors:** Krzysztof Miler, Karolina Kuszewska, Gabriela Zuber, Michal Woyciechowski

**Affiliations:** Institute of Environmental Sciences, Jagiellonian University, Gronostajowa St. 7, 30-387 Kraków, Poland

**Keywords:** Antlion, Behavioural asymmetry, Brain lateralization, Cognitive skills

## Abstract

Recently, antlion larvae with greater behavioural asymmetry were shown to have improved learning abilities. However, a major evolutionary question that remained unanswered was why this asymmetry does not increase in all individuals during development. Here, we show that a trade-off exists between learning ability of larvae and their hunting efficiency. Larvae with greater asymmetry learn better than those with less, but the latter are better able to sense vibrational signals used to detect prey and can capture prey more quickly. Both traits, learning ability and hunting efficiency, present obvious fitness advantages; the trade-off between them may explain why behavioural asymmetry, which presumably stems from brain lateralization, is relatively rare in natural antlion populations.

**Electronic supplementary material:**

The online version of this article (10.1007/s10071-018-1190-2) contains supplementary material, which is available to authorized users.

## Introduction

Many animals respond differently to stimuli on one side of their body than they do to stimuli on the other. Such behavioural asymmetry presumably reflects the level of brain lateralization, i.e., brain functions that involve one hemisphere more than the other (Rogers et al. 2013). In humans and other vertebrates, the connection between the behavioural asymmetry and the brain lateralization is widely assumed (Levy 1977) and has some experimental support (Vallortigara and Rogers 2005). For instance, the increased ability to simultaneously perform two tasks (predator vigilance and food searching) has been demonstrated to be associated with high brain lateralization in chicks (Rogers et al. 2004). This connection has also been demonstrated in invertebrates, such as in fruit flies, which show superior memory when they possess highly lateralized brains (Pascual et al. 2004). More frequently, however, the level of brain lateralization is simply inferred from the level of behavioural asymmetry. In fish, several cognitive advantages of being highly behaviourally asymmetric (and presumably having high brain lateralization) have been demonstrated (see Sovrano et al. 2005 and, more recently; Bibost and Brown 2014; Dadda et al. 2015). In invertebrates, too, behavioural asymmetry was shown to increase cognitive functioning, i.e., learning speed (see Miler et al. 2017 for an example in the predatory neuropterans, antlions), possibly reflecting the benefits of the brain lateralization.

In predatory species, the detection of stimuli that co-occur with prey encounters, such as visual cues, enable the anticipation of prey arrival, thus increasing capture success. In antlions, vibrational cues correlated with prey arrival can be learned and used to modify foraging strategy in adaptive ways (Kuszewska et al. 2016), so these organisms should be selected for more efficient learning and thus greater fitness. However, only 24% of *Myrmeleon bore* antlions originating from a single population in Poland were reported to show increased levels of behavioural asymmetry, which, as mentioned above, correlates with their enhanced cognitive performance (Miler et al. 2017). From an evolutionary perspective, this finding indicates potential major fitness costs of behavioural asymmetry in certain kinds of tasks. For example, antlions live in sandy areas and capture prey using pitfall traps (Scharf et al. 2011), and tossing sand at a prey item is a tactic that can increase capture success. However, the efficiency of this behaviour depends on the direction in which the sand must be thrown (Bongers and Koch 1981) and thus may differ due to individual behavioural asymmetry (i.e., side bias).

Here, we used *M. bore* antlions with higher or lower behavioural asymmetry to test the hypothesis that more-biased individuals perform better at a cognitive task but worse at hunting prey than less-biased individuals. Interestingly, behavioural asymmetry was observed previously in the context of foraging (for examples on toads see Vallortigara et al. 1998 and Robins and Rogers 2004) but not in connection to learning.

## Methods

We collected 200 *M. bore* larvae from the Błędowska Desert (Poland) and assessed their preferred direction in righting behaviour in 20 trials (allowing ~ 10 min between trials) (Miler et al. 2017). In each trial, a single larva was placed inside a plastic Eppendorf tube (1.5 ml) that was then gently shaken. This resulted in the larva falling on its back, and we noted the direction (left or right) in which it righted itself. Then, we categorised the larvae into two groups: (1) more lateralized (left turns occurring in 0–5 or 15–20 trials) and (2) less lateralized (left turns occurring in 6–14 trials). Afterwards, each larva was weighed, and 24 weight-matched groups of 4 larvae each were created, with each group comprising two more and two less lateralized individuals (96 larvae in total). These larvae were individually housed in paper boxes (25 × 15 × 10 cm) that were half-filled with sand, fed a single ant prey item (live *Lasius niger* worker) and left to acclimate and build traps for 48 h. Within each group, one more lateralized and one less lateralized individual were assigned to the relevant (contingent) condition, and the other two larvae were assigned to the irrelevant (non-contingent) condition. Two groups of larvae (eight individuals) were excluded because some of the individuals within these groups failed to build traps. In total, we tested 88 larvae, 44 in the relevant and 44 in the irrelevant condition (22 more lateralized and 22 less lateralized individuals in each case).

The experiment was run in blocks, each involving 2 days of training followed by a break day. Ants were placed in antlion pits twice per training day, between 10 a.m. and 6 p.m. Larvae in the relevant condition were presented with a vibrational cue approximately 10 s before prey delivery, whereas larvae in the irrelevant condition were presented with the vibrational cue 5–10 min before or after prey arrival, thus providing no opportunity to associate the cue with the prey. Vibrational cues involved the delivery of 4.5 ml of sand through a funnel with an attached plastic pipette tip directed towards the edge of the antlion pit, and a small container (a metal pipe, 4 cm in diameter, blocked off at the bottom with a sheet of foil) below the pipette prevented additional sand from accumulating in the box and enabled vibrations to be conducted (see Supp. Fig. 1A). Each delivery of the vibrational cue to an antlion was treated as a test. Larvae prepare for prey arrival (i.e., show mandible movement at the bottom of the pit) when they make the association with the vibrational cue. Therefore, after cue delivery but before prey delivery (as mentioned, ~ 10 s), preparation for hunting (i.e., reaching the learning criterion) can be easily observed. Once an individual showed mandible movement after the cue in two consecutive tests, it was marked as having reached the learning criterion. The following day, the distance at which the vibrational cue elicited the learned response in these individuals was tested (the distance test). For each larva, six distances from the edge of the antlion pit were used, in decreasing order, with a 10-min interval between the different testing distances: 15, 12, 9, 6, 3 and 0 cm. Vibrational cues were delivered at these distances as 4.5 ml of sand falling from a funnel with a plastic pipette tip into a small container below. Prey was never delivered during the distance test. Since they did not learn, the larvae in the irrelevant condition never proceeded to the distance test. Hence, in each group of four larvae, the training sessions for the two larvae in the irrelevant condition were terminated when both larvae in the relevant condition reached the learning criterion. The next day, prey capture latency was tested in the larvae from the irrelevant condition (the latency test). A circular plastic arena (11 cm in diameter) covered in Fluon (Sigma–Aldrich, Germany) was placed around the antlion pit, and a group of five live *L. niger* worker ants was introduced (see Supp. Fig. 1B). The test began when the first worker stepped into the antlion pit, and we measured the latency (in seconds) to the capture of any of the ants. The maximum test time was 3 min.

Statistical analyses were conducted in STATISTICA 13 (Tibco, Poland). The learning speed of the larvae in the relevant condition, with group (more vs. less lateralized) as a factor, was analysed using the Wilcoxon matched-pairs test (dependent variable: the number of sessions to reach the learning criterion). The maximum distance at which the vibrational cue elicited the learned response in the larvae in the relevant condition in the distance test (dependent variable: the distance at which the learned response was evident) and the latency to prey capture in the larvae in the irrelevant condition in the latency test (dependent variable: the latency to ant capture) were analysed similarly.

## Results

**Fig. 1:**
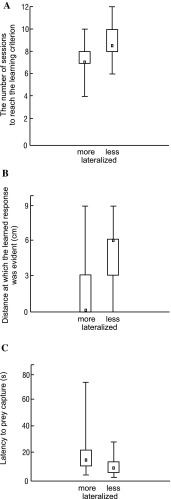
Behaviour of more and less lateralized antlion larvae. **a** The number of sessions to reach the learning criterion in the relevant condition. Valid *N* = 22 pairs; *Z* = 3.944; *p* < 0.001. **b** Distance from the edge of the pitfall trap at which the learned response was evident. Valid *N* = 22 pairs; *Z* = 3.506; *p* < 0.001. **c** Latency to prey capture. Valid *N* = 22 pairs; *Z* = 3.685; *p* < 0.001. Statistics: Wilcoxon matched-pairs tests. Squares indicate medians, boxes indicate quartiles, and whiskers indicate ranges

We detected 48 highly asymmetric individuals out of a total of 200 larvae tested for bias in righting (24%). None of the larvae in the irrelevant condition “learned” the focal association, which was not surprising, as this condition was designed solely to ensure that only individuals in the relevant condition learned. The occurrence of a learned response was significantly lower in terms of the number of sessions required to reach the learning criterion among more lateralized larvae than less lateralized ones (Fig. 1a), indicating that the former learned more quickly than the latter. However, the occurrence of a learned response was significantly lower in terms of the maximum distance at which the response was evident among more lateralized larvae than among less lateralized ones (Fig. 1b), meaning that the more lateralized larvae showed lower sensitivity to vibrations than the less lateralized larvae. Furthermore, the latency to ant capture was significantly higher among the more lateralized larvae than among the less lateralized ones (Fig. 1c), indicating that the more lateralized individuals showed lower hunting efficiency.

## Discussion

Our data show that antlion larvae that display behavioural asymmetry learn faster, but they simultaneously experience decreased vibration sensitivity and, probably as a result of this, exhibit lower hunting efficiency. These results strongly suggest that a trade-off exists between larval ability to hunt efficiently and learn quickly. In this study, we detected the same ratio (24%) of highly asymmetric individuals in a population as in the previous study reporting asymmetry in *M. bore* and utilising the same type of side-bias testing (Miler et al. 2017). This low number is much more likely to be connected with the trade-off than with the methods employed (i.e., the categorization of individuals as more or less lateralized on the basis of a single behavioural measure), as previously suggested (Miler et al. 2017).

Fitness costs have been found to be associated with asymmetry in other animals. Fish pay these costs when they are faced with tasks requiring matched information from both sides of the body (Dadda et al. 2009) and when forced to compete for resources (Chivers et al. 2017), whereas behaviourally asymmetric dogs experience problems when solving puzzles (Marshall-Pescini et al. 2013). In antlions, the costs of behavioural asymmetry in terms of lower hunting efficiency may stem from lower vibration sensitivity, at least partially connected to morphological asymmetry at the peripheral level, as was demonstrated for several species of bees in connection to their learning abilities (Anfora et al. 2011; Frasnelli and Vellortigara 2017).

Importantly, there is an alternative interpretation of the results of the distance test. Here, larvae were trained to associate the cue with the prey at the edge of their trap and then tested for the learned response at several distances from their pitfall trap in the distance test. Vibrations delivered farther away from the larvae differ in strength from the learned cue. Thus, it may be that the more lateralized larvae show less generalization (Shepard 1987; Ghirlanda and Enquist 2003). In this context, it is not a bad thing, because vibrations may occur not only due to the approaching prey but also due to various distractors. Subsequently, showing hunting readiness to all vibrations would be unnecessary. In any case, the issue of vibration sensitivity in the more and the less lateralized groups of antlions seems worth further study as it may be quite the opposite from suggested above: if the more lateralized group perceives the learned and tested stimuli as more different (i.e., shows less generalization) than the less lateralized group, then the former should have higher vibration sensitivity. The distance test, then, leaves us no hint as for the reasons behind differences in hunting efficiency between more and less lateralized larvae.

Our results are consistent with previous reports that antlions can associate vibrations with environmental events (Guillette et al. 2009; Hollis et al. 2011; Kuszewska et al. 2016) and that those with pronounced behavioural asymmetry possess superior cognitive skills (Miler et al. 2017); here, this latter phenomenon was demonstrated with a novel task (i.e., hunting readiness as opposed to prey burial in the previous study). The hypothesis that behavioural asymmetry conveys fitness advantages, especially in a cognitive context, is gaining experimental support (Güntürkün et al. 2000; Magat and Brown 2009), but the evidence is still scarce for invertebrate species. An important step that is missing here is the demonstration of the direct connection between the behavioural asymmetry and the brain lateralization, presumably responsible for behavioural side bias (Miler et al. 2017).

Overall, we demonstrate that behavioural asymmetry is associated with superior cognitive and inferior hunting performance in larval antlions. A trade-off between these two traits might explain why brain lateralization is relatively rare in natural antlion populations.

## Electronic supplementary material

Below is the link to the electronic supplementary material.

Supplementary material 1 (XLSX 11 KB)

Supplementary Figure 1. Schematic representations of the experimental setup. A) The paper box (25 × 15 × 10 cm) half-filled with sand and an antlion housed inside, present at the bottom of its pitfall trap. Funnel with an attached plastic pipette tip used for delivering sand into the small container placed at the edge of the pitfall trap. The movable holder of the funnel allowed sand delivery at different distances from the edge of the pitfall trap in the distance test (0, 3, 6, 9, 12 and 15 cm). B) The same paper box arranged for the latency test. The funnel and its holder, as well as the container, are absent. Instead, a circular plastic arena (11 cm in diameter) covered in Fluon is placed around the trap, and 5 worker ants are introduced (EPS 296 KB)
